# Supplementary material for: Data-driven contact structures: From homogeneous mixing to multilayer networks
Source: PLoS Comput Biol. 2020 Jul 16;16(7):e1008035. doi: 10.1371/journal.pcbi.1008035 (PMC7386617; doi:10.1371/journal.pcbi.1008035)
Supplement: Attachment — Submitted filename: ResponseReviewers.pdf [file pcbi.1008035.s001.pdf]

Reviewer #1:

In "Data-driven contact structures: from homogeneous mixing to multilayer networks" authors bridge for the first time an important gap that has existed until now between data analysis and contact structures derived from this data, namely the simultaneous determination of both, the connectivity of individuals and the age-structure of the population. This advance has important implications for the mathematical modeling of infectious diseases, as beautifully shown in the paper. In particular, authors compare four different scenarios that gradually leads to taking into account a multilayer representation in which both the social mixing and the number of contacts are included in the model. It is shown analytically that the thresholds obtained for these four scenarios are different, and that indeed only the most comprehensive framework, as presented in the paper, allows for the correct determination of the epidemic threshold. This is further supported by systematic simulations, confirming further that heterogeneities in the contact network are vastly important and must not be overlooked if we wish for a proper determination of the epidemic threshold. It is also shown that the age-structure, which is likewise determined by the new approach, plays a bigger role beyond the onset of the outbreak.

An accurate determination of epidemic thresholds in contact networks is of huge importance, both for mitigating and prediction epidemic spreading, as well as for devising effective vaccination strategies. This research points out clearly for the first time that, when it comes to the evaluation of interventions such as vaccination, both sources of individual heterogeneity are important and should be considered jointly. This was an important open problem in the realm of an intensely investigated subject with obvious practical ramifications. By introducing a clever new approach based on empirical data and network science, this study thus fills and incredibly important gap that bridges the divide, and it reveals just how wrong one could be by neglecting or not having access to all the needed information in terms of connectivity and age of the population.

The paper is well-written, comprehensive, and clear. I find it is among the finest papers that I have had the pleasure of reading in the recent past. The motivation behind the approach and the insights it affords towards improving spreading of communicable diseases is genius, and as such it will surely not fail to impress the diverse readership of PLOS Computational Biology. For these reasons, I warmly recommend publication.

It is quite a challenge to suggest improvements for such an excellent contribution. Perhaps a reference to the current COVID-19 pandemic and how the approach could improve forecasting, as studied in "Forecasting COVID-19", Front. Phys. 8, 127 (2020), would be worthwhile. Apart from this, I can only reiterate my overall very positive impressions and congratulate the authors to a fine contribution.

We sincerely thank the reviewer for the very positive assessment of our work.

Following his/her advice, we have added a brief comment in the introduction and a few lines regarding the current COVID-19 pandemic in the conclusions section. We sincerely thank the reviewer for pointing this out because we indeed believe that the modeling of this disease would particularly benefit from our results. Furthermore, the current lack of data regarding the mixing patterns in countries such as Spain shows that this data's importance is usually neglected. We hope that our paper will help to raise the interest of the community in this information.

Reviewer #2:

This manuscript proposes a model which considers the aspects (i) the number of contacts of individuals and (ii) age-dependent contact pattern. Four different versions of the model are introduced depended on whether considering the heterogeneity of the two aspects. The performance of the models is examined with SIS and SIR epidemic models based on a data of human contact in Italy. I think this proposed model is interesting. However, this work looks only on its half way which demands a more systematical evaluation under a wide parameter space. The main results presented in the work are based on a specific data and the provided conclusions may not show its generality. Thus, I could not recommend its publication on Plos Computational Biology.

Following are some points for the notice of the authors:

1. The most results shown in this manuscript are based on the parameters induced from the data of Italy. A systematic investigation of the model is recommended from which general properties pertain to the model need to be abstracted and the results of empirical networks could serve as a potent examples.

We agree with the reviewer in the fact that we have only used one set of data. However, we do not think that extending the analysis to other countries will add much information. In particular, the most detailed data on this regard comes from the POLYMOD study, which was performed in Europe. As such, the mixing patterns and the contact distribution of the population is fairly similar from country to country. It is true that the precise values might change, for instance the average degree in Italy is 19, while in Germany it is 9. Yet, although the exact values of the attack rate - and other analysis - will change, the shape of the contact patterns matrix and the demography are fairly similar. Thus, the qualitative results will be the same. Since we are focused precisely on a qualitative analysis of the role that this kind of data plays, adding more countries will obscure the discussion. Note, additionally, that although we used a dataset for Italy, the methodology is general and not specific to this data set.

2. Many concepts and legends in the figures are not well defined. For example, the "Frequency" in Fig. 1D; and is the "number of contacts" should be "age"? What is the 'X' in Fig. 2B? What is the "Relative Difference (%)" in Fig. 2C.

We have added a sentence in the caption to better explain the meaning of figure 1D. We understand that there might be some confusion regarding the term “frequency” since sometimes it is used as the total count while in others - like in our case - is normalized. Regarding the x axis, the number of contacts is the correct term. In the plot we show the number of contacts that any individual has, ranging from between 0 and 5 contacts to over 45 contacts per day. We hope that the new sentence in the caption clarifies this.

In figure 2B, X represents the susceptibility. We have rephrased the sentence citing this figure to make this clearer. Besides, we also discussed this measurement in the Materials and Methods section.

Lastly, in figure 2C, as explained in the caption, the y axis represents the relative difference in the number of infected individuals between the results obtained using each method and the homogeneous setting. Thus, it is the difference between the number of infected individuals using one method and using the homogeneous mixing over the value of the homogeneous mixing. In other words, we are using the relative difference.

3. Some results in the figures seem not sufficient in its reliability. For example, generally, human contacts are symmetric and reciprocal. However, the gray plot in Fig. 1C looks asymmetric.

We are not sure whether we understood this comment by the reviewer. Please, note that as we mention in the text, the more general situation corresponds to asymmetry. Indeed, figure 1C is asymmetric. This was explained in the paragraph starting in line 176. These matrices would be symmetrical only if the demographic distribution of the population were homogeneous. Suppose that we have a population of 2 individuals, each of them having one link towards a third individual who constitutes its own group. The matrix representing these interactions would be

(0,1)
(2,0)

So that M_{1,2} != M_{2,1}. Yet, the relation M_{1,2} N_1 = M_{2,1} N_2 will hold, due to the different sizes of the groups (N_1=2, N_2=1). This was explained in equation (2) and line 180.

4. In addition, in Fig. 3B, what is the definition of R0 according to the age and more importantly how is this age-dependent R0 obtained. Are the results in Fig. 3B theoretical or numerical. If numerical, what is the number of realizations in the simulation and what are the sizes of error bars?

As explained in line 270, we count the number of newly infected individuals that a single infectious subject would produce in a fully susceptible population. To obtain the R_0 for each age group, as explained in line 274, we measure this value as a function of the seed node's age. We have clarified that the result comes from the numerical simulation by adding that they were obtained after 10^8 runs. This is also the reason why we have not included error bars since the error on the estimation of the mean is negligible with such a large number of simulations.

5. The results in Fig. 2C seem to be obtained from the contact pattern in Fig. 1C. However, in the manuscript it is not sufficiently clarified. Since this result presented together with A and B, confusion is easily occurred.

In the three panels of figure 2, we always use the four models: homogeneous (H), social mixing (M), contacts (C), and multilayer (CM). Each of them uses a different set of data, as explained in figure 1. We keep the same notation throughout the document, so we believe it is clear which set of data we are using at each step.

6. Why the study of vaccination is only put on SIR model? Since the SIS model is also an object being addressed in this work, the study of the vaccination should also been applied to the SIS model for the completeness of the work.

The Susceptible-Infected-Susceptible model represents disease dynamics in which the individual can only be in two possible states: healthy (susceptible) or infected (and infectious). It does not allow for individuals that have acquired immunity, either naturally after recovering from the disease or via vaccination. This is the reason why we restrict our analysis to an SIR model.

7. Some arguments are difficult to understand, e.g. "To gauge the effect ..." in line 300.

We are not sure whether we understood this criticism. “To gauge the effect” is standard english and it means “to estimate/assess/evaluate the effect”.

8. As mentioned, the results of this manuscript are largely based on a specific data. Therefore, the observations from the data may not suggest a general conclusion. Thus, I don't think the arguments under general terms without mentioning specific conditions, for example "This result might seem ..." in line 312, could explain the specific observations on its above, since in other conditions the observations could be essentially different.

We agree that with very different mixing matrices and contact distributions, the results could be different. That is the reason why we are proposing this methodology. The paper's purpose is to show some of the effects that this methodology might have. Here we focused on Italy's case, just an example of an application. Since the matrices, distributions, vaccination rates, and

demography vary both in time and space, different results are expected. However, this does not diminish the conclusion: it is essential to account for both the contact distribution and the age mixing patterns of the population.

9. "Attack rate" looks not a common term used in the complex network epidemiology.

We thank the reviewer for this comment. We have added a sentence clarifying its meaning in the manuscript. We hope that this might help us to appeal to a broader audience.
